# Current Occurrence of the Atlantic Sturgeon *Acipenser oxyrinchus* in Northern Spain: A New Prospect for Sturgeon Conservation in Western Europe

**DOI:** 10.1371/journal.pone.0145728

**Published:** 2015-12-30

**Authors:** Benigno Elvira, Sheila Leal, Ignacio Doadrio, Ana Almodóvar

**Affiliations:** 1 Department of Zoology, Faculty of Biology, Complutense University of Madrid, Madrid, Spain; 2 Department of Biodiversity and Evolutionary Biology, National Museum of Natural Sciences, CSIC, Madrid, Spain; Institute of Marine Research, NORWAY

## Abstract

*Acipenser oxyrinchus* is considered extirpated in Europe, but numerous breeding populations still exist on the Atlantic coast of North America. An adult female *A*. *oxyrinchus*, 2500 mm total length and 120 kg wet weight, was accidentally fished on 24 November 2010 near the coast of Gijón, Asturias, Spain. The fish was identified by its morphological pattern as well as by mitochondrial and nuclear DNA analyses. Because the sturgeon was found far away from any known breeding area, it was considered a stray or vagrant specimen. It certainly has a natural origin, but its eventual birthplace could not be determined. Because its current occurrence was unknown in southwestern Europe until now, the species is not cataloged or protected in this area. Therefore, the residual European stocks of *A*. *oxyrinchus* ought to be listed as Critically Endangered (CR) according to the IUCN categories. Likewise, it is imperative for southwestern European countries with an historic or recent occurrence of *A*. *oxyrinchus* to protect the species through domestic and international legislation. The present sympatric occurrence of *A*. *sturio* and *A*. *oxyrinchus* raises new challenges about key questions, such as the species selection for restoration program in European countries. Accurate monitoring is mandatory to obtain appropriate information for an assessment of the current occurrence of *A*. *oxyrinchus* in southwestern Europe.

## Introduction

The family Acipenseridae consists of 25 anadromous and freshwater sturgeon species of circumpolar distribution in the Northern Hemisphere. Unfortunately, sturgeons are seriously threatened due to overfishing, damming of rivers and pollution [1–3]. Three species lived in southwestern Europe: the European sturgeon *Acipenser sturio*, the Atlantic sturgeon *Acipenser oxyrinchus* and the Adriatic sturgeon *Acipenser naccarii* [4].

Traditionally, only *A*. *sturio* was considered to have lived in northeastern Atlantic coasts and rivers [5]. However, taxonomic (specific) differences between northern (Baltic Sea) and southern European Atlantic populations have been reported [6–8]. In contrast, *A*. *oxyrinchus* was considered to live only along the eastern coast of North America [9].

Natural occurrences of *A*. *oxyrinchus* in the Baltic Sea since the Middle Ages have been reported based on genetic, morphological and archaeological evidence, which indicates that *A*. *oxyrinchus* colonized the Baltic Sea between 1200 and 800 years ago, when it replaced the formerly native sturgeon species there, *A*. *sturio* [10]. This would have been a unique case of a natural transatlantic trip for a freshwater-spawning fish. Later, it was proposed that *A*. *oxyrinchus* introgressed into, rather than replaced, the *A*. *sturio* population in the Baltic Sea [11]. The eventual genetic mosaic pattern found in the Baltic sturgeon population could have been caused by a sex-biased introgression where spawning was largely restricted to immigrating American *A*. *oxyrinchus* females and where fertilization was predominantly achieved by abundant local European *A*. *sturio* males. This hybrid nature of the Baltic sturgeon population was refuted, however, using both mitochondrial and nuclear markers, proving that a short number of the northernmost (Canadian) specimens of *A*. *oxyrinchus* were the original founders of the recent Baltic sturgeon population [12].

Sturgeon remains from several archaeological sites along the French Atlantic coast showed that *A*. *oxyrinchus* was already present in that region at the end of the fourth millennium BC [13–15]. An accurate revision of further French archaeological sites demonstrated the sympatric occurrence of *A*. *sturio* and *A*. *oxyrinchus* for a long time in the Atlantic drainages and the French Atlantic coast until the 17th century [14–17]. Molecular evidence of sympatry and natural reciprocal hybridization between *A*. *sturio* and *A*. *oxyrinchus* on the French Atlantic coast during the 19th century was also presented [17].

The sturgeon *A*. *oxyrinchus* probably arrived in Europe after the Last Glacial Maximum [17]. A colonization of the French coasts by *A*. *oxyrinchus* prior to the establishment of the Baltic population 1200 years ago is therefore likely, as corroborated by its presence in the area from the end of the fourth millennium BC [15]. However, the time of entry of *A*. *oxyrinchus* into the Baltic Sea was later estimated to have been 4000–5000 years ago [18].

On the other hand, the earliest known evidence for the existence of *A*. *oxyrinchus* in British rivers came from two specimens caught during the 19th century in Yorkshire, northeastern England [19]. The last known record of this species from the UK is a large specimen caught in Wales in 2004 [20]. Furthermore, the occurrence of an *A*. *oxyrinchus* specimen was reported in the Netherlands in the 19th century [21].

Although *A*. *oxyrinchus* is considered extirpated in Europe [22], numerous breeding populations still exist on the Atlantic coast of the United States and Canada [23], where two subspecies are recognized: *A*. *oxyrinchus oxyrinchus* occurs in rivers from the St. Lawrence River in Canada to the St. Johns River in Florida, and the Gulf Sturgeon *A*. *oxyrinchus desotoi* lives in Gulf of Mexico drainages from the Apalachicola River in Florida to the Mississippi River in Louisiana [24].

Currently, in southwestern Europe, only relict sturgeon populations remain in the Garonne River basin in France (*A*. *sturio*) [25] and in the Po River basin in Italy (*A*. *naccarii*) [26]. The goal of this paper is to report the first natural recent occurrence of *A*. *oxyrinchus* in southwestern Europe and to discuss the new prospects and challenges for sturgeon recovery in the area in light of this finding.

## Material and Methods

### Material

An adult sturgeon specimen was accidentally caught by commercial fishermen using a small boat with a trammel net at approximately 20:00 (local time) on 24 November 2010. The catch took place near the coast of the San Lorenzo beach (“Playa de San Lorenzo”), Gijón, Asturias, Spain (43° 33' 05'' N, 5° 38' 50'' W; UTM 30T2864825). The fish was caught at a depth of approximately 8 m, over a sandy bottom. The dead specimen was landed in the nearby port of “El Musel”, Gijón, carried to the Gijón Aquarium and later taken to the Center for Fishing Experimentation, where it was immediately frozen. The fishermen were not allowed to catch sturgeon but, in fact, its accidental capture is not illegal according to the domestic fisheries regulations. The regional Directorate General of Biodiversity and Landscape, the competent authority in protected species (supposing the specimen belonged to the protected species *A*. *sturio*), took over the fish, which was deposited in a public collection. Unfortunately, the regulations regarding accidental sturgeon catches in Spain are not enough yet to protect these solitary specimens. Sturgeons are generally unknown by fishermen and they are not aware of the species' legal status and of the instructions to be followed if caught. One of the reasons for writing this manuscript has been to alert on these facts.

We only could analyse the fish after its death and landing. Thus, the thawed specimen was studied by one of the authors (B.E.) when it was defrosted on 26 November 2010 and 11 May 2011. The specimen had no visible tags on its body or in the visceral cavity. The specimen was weighed, dissected, sexed, and the gonads and liver were weighed. The condition factor (100 x the total body weight in grams / total length cubed in cm), gonadosomatic index (100 x gonad weight / total body weight) and hepatosomatic index (100 x liver weight / total body weight) were calculated. Samples of muscle were dissected and preserved in 100% ethanol for molecular analyses. The specimen was then fixed in formalin, preserved in 70% ethanol and displayed in the exhibitions of the Aquarium—Museum of the Centre for Fishing Experimentation, Directorate General for Fisheries, Government of the Principality of Asturias, at Gijón, Spain. The specimen was cataloged with the code number CEP 20101125–001.

### Methods

#### Morphology

Several diagnostic morphological, morphometric and meristic characters [7–8, 14–15, 22, 27–30] were used. Thirty morphometric and ten meristic characters were determined [8, 28, 31]. The presence and shape pattern of the bony plates and denticles was also researched. The specimen was also checked for the presence of a fontanel between the frontal and parietal bones, which is typical for *A*. *oxyrinchus* juveniles [22].

#### Molecular analyses

Total DNA was extracted from a fresh muscle tissue sample using a DNeasy Tissue Kit (QIAGEN, IZASA, Barcelona, Spain). Molecular markers included microsatellite nuclear loci, together with markers for the complete mitochondrial regions of the cytochrome *b* (Cyt b) and D-loop genes.

Five microsatellite loci were analyzed: *LS19*, *LS54*, *LS68* [32], *Aox23* [33] and *AoxD161* [34]. These nuclear markers were selected because they allow the distinction among the three sturgeon species that may eventually occur in southwestern Europe [4]. Details on PCR conditions, primer pairs and interpretations of microsatellite polymorphisms were performed as reported in [4]. PCR products were analyzed using an ABI PRISM 3730 Genetic Analyzer (Applied Biosystems, CA, USA). Allele sizes were scored using Peak Scanner^™^ Software v.1.0 (Applied Biosystems).

The entire Cyt b was amplified according to [35]. The D-loop was amplified using the primer HeteroI (*5′*-ACCCTTAACTCCCAAAG-3′) developed by [36] and a new primer (dloop-rev: *5′*-CATCTTCAGTGTTATGCTT-3′) located in the phenylalanine tRNA gene that flanks the control region.

Polymerase chain reactions (PCR) were performed in a final volume of 50 μl containing 100 ng genomic DNA template, 0.4 mM of each primer, 0.4 mM of dNTPs, 1X PCR buffer included 2.5 mM MgCl_2_, and 1 unit of Biotools HotSplit DNA polymerase (Biotools, Madrid, Spain). The thermocycle profile consisted of 94°C for 5 min, 30 cycles of 94°C 1 min, 50°C 30 s, and 72°C 90 s, with a final extension at 72°C for 10 min. The sequence reaction products were then analyzed using an ABI Prism 3730 Genetic Analyzer (Applied Biosystems, CA, USA).

Sequences were then compared with known sturgeon sequences in GenBank using the BLAST search algorithm [37] and aligned with CLUSTALX [38]. Pairwise distances between nucleotide sequences were computed according to Kimura’s two-parameter model, using the program MEGA v. 6.0 [39]. The D-loop complete sequence was deposited in the GenBank database under the accession number KT007224.

#### Taxonomy

*Acipenser oxyrinchus* is currently recognized as a valid species distinct from *A*. *sturio* [4, 40]. Problems with nomenclature of these two sister species were recently resolved [29], fixing *A*. *oxyrinchus* as the name of the Baltic and North American sturgeon species and *A*. *sturio* as the name of the western and southern European species. A neotype for *A*. *sturio* was also designated [29], whereas no types are known for *A*. *oxyrinchus*.

## Results

### Morphology

**Fig 1:**
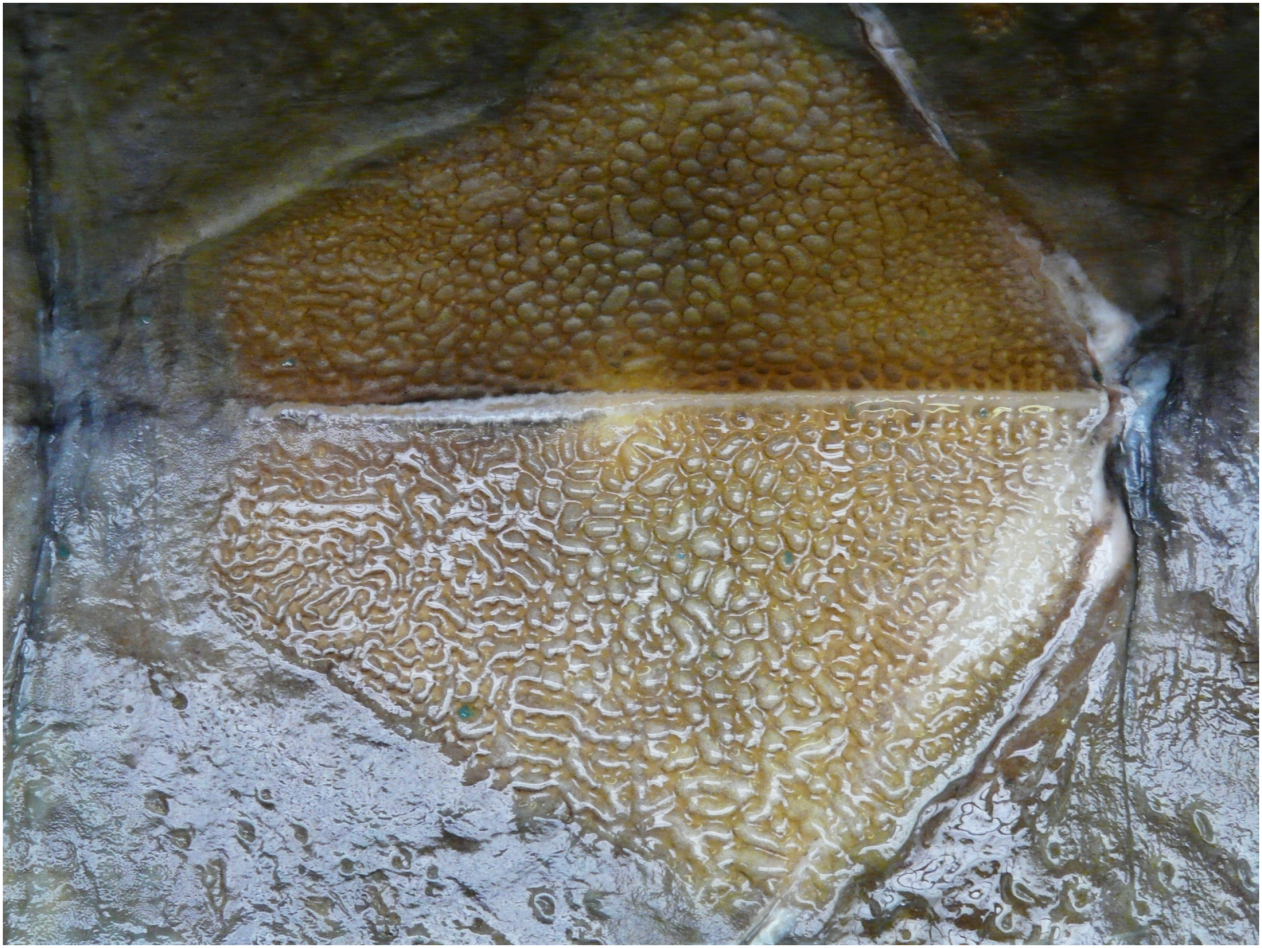
Dorsal scute view. Second dorsal scute of the sturgeon specimen caught off the coast of Gijón in 2010, presenting on its surface deep and circular alveoli, separated by thin septa, typical of *A*. *oxyrinchus* (Photo B. Elvira, 11 May 2011).

**Fig 2:**
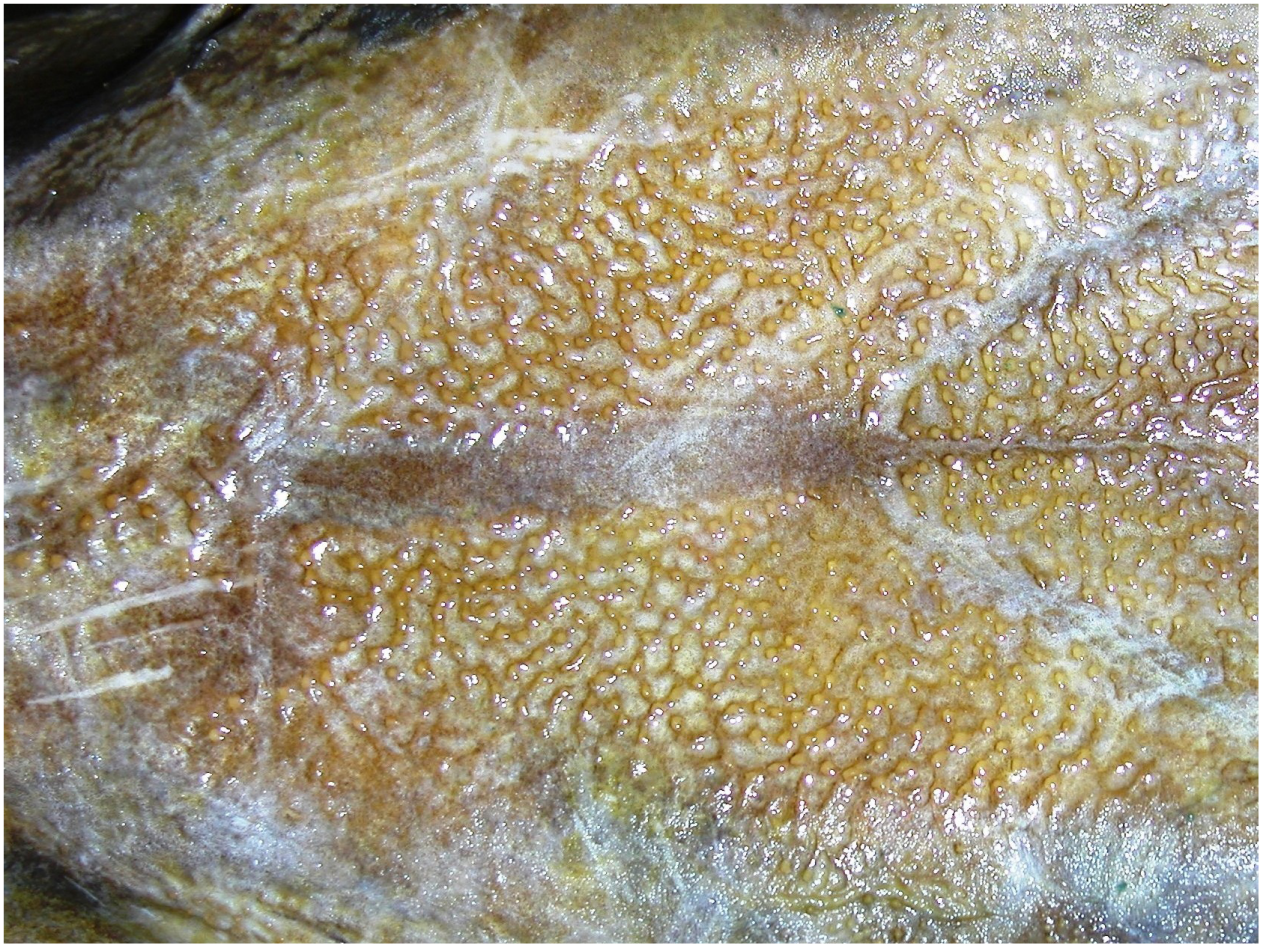
Partial dorsal view of the head. A pineal bone fills the fontanel space and is clearly visible between the frontal bones, anteriorly to the parietal bones (Photo B. Elvira, 11 May 2011).

Morphometric and meristic characters of the sturgeon specimen caught off the coast of Gijón in 2010 are included as Supporting Information (S1 Table). The nuchal or postoccipital bone (sometimes considered as the first dorsal scute) is fused with the dermocranium behind the supraoccipital bone. Some plates are present on the left and right side of the dorsal and anal fin base. The laterodorsal and lateroventral surface of the body is densely armored with rhombic plates arranged in oblique rows. The specimen shows a typical alveolar pattern on the surface of its large scutes (Fig 1). The small rhombic plates on the laterodorsal body surfaces also display these characteristic differences. A pineal bone fills the fontanel space and is clearly visible between the frontal bones, anteriorly to the parietal bones (Fig 2).

The dorsal part of the body is brownish green; the coloring is less intense on the sides, and becomes white on the ventral surface. The tip of the snout shows a black patch. The peritoneum is pale.

The specimen is a female with a wet weight of 120 kg. The gonad weight was 1879 g and liver weight was 2454 g. The condition factor was 0.768, gonadosomatic index was 1.565 and hepatosomatic index was 2.045.

### Molecular analyses

The five microsatellite loci studied in the Spanish sturgeon specimen show allelic patterns compatible with a disomic inheritance. These results reject the possible identification of the specimen as *A*. *naccarii* because this species has tetrasomic patterns for four of the five loci [4]. The alleles for *LS54* (116) and *Aox23* (104) of the specimen are included within the allele ranges known for *A*. *oxyrinchus* and excluded from those of *A*. *sturio* and *A*. *naccarii* [4] (S1 Fig). These two alleles allow the sturgeon from Gijón to be identified as *A*. *oxyrinchus*. Furthermore, the loci *LS68* and *AoxD161* show allele sizes within the defined range for *A*. *oxyrinchus* and *A*. *naccarii*. Finally, the locus *LS19* presents two allelic variants, one of which (146) prevents identification of the sample as *A*. *naccarii*.

The entire Cyt b and D-loop genes were successfully amplified in the Spanish sturgeon specimen. The Cyt b (1139 bp) and D-loop (880 bp) sequences were aligned with those of *A*. *oxyrinchus* and *A*. *sturio* retrieved from GenBank (S2 and S3 Tables). The Cyt b sequence from the specimen is identical to the *A*. *o*. *oxyrinchus* haplotypes found on both sides of the Atlantic Ocean, as well as in *A*. *o*. *desotoi* [18]. Likewise, the entire D-loop sequence from studied specimen does not show any variable site with the short fragment described as the haplotype A of the *A*. *o*. *oxyrinchus* widespread in the western Atlantic Ocean and Baltic Sea (historically and presently) [12, 18, 41–42].

In sum, the Cyt b and D-loop gene sequences are congruent with the nuclear ones and clearly identified the specimen with the most common haplotypes of *A*. *oxyrinchus*.

### Taxonomy

The sturgeon specimen caught off the coast of Gijón in 2010 was preliminarily determined as Atlantic sturgeon *Acipenser* sp. [43]. After our analyses, which were based on morphological characters and molecular markers, the specimen was definitively determined to be *A*. *oxyrinchus*, being the first known record of the species in the Bay of Biscay, northern Spain. Likewise, it represents the first recent occurrence of the species in southwestern Europe, when this species was supposed to have been extirpated in Europe [22], with the formerly last known record of this species being a sturgeon caught off the Welsh coast in 2004 [20].

### Origin

The eventual origin of sturgeon caught near Gijón is unclear, because the molecular analyses revealed that it bears two mitochondrial haplotypes (Cyt b and D-loop) that are common in northern Europe as well as in the northernmost area of Canada and the United States. However, the entire Cyt b gene sequence from the specimen shows one bp change when aligned with the fragment (344 bp) coming from the specimens stocked in Polish waters (sequence deposited in Gene Bank under accession number KC987018) [44].This fact supports the possible natural origin of the sturgeon caught in Spain.

## Discussion

### Morphology

Morphological diagnostic characters support the identification of the sturgeon caught at Gijón as being *A*. *oxyrinchus* [22]. For example, the surface appearance of large scutes is alveolar (has small holes) indicating *A*. *oxyrinchus*, whereas tuberculata (small protuberances) would have indicated *A*. *sturio* [13–14]. With regard to the other diagnostic character [22], no fontanel, as observed in *A*. *oxyrinchus* juveniles, was present, but a pineal bone closes the fontanel space between the frontal bones, which typically occurs in very large adults [45].

The number of bony dermal scales of the dorsal, lateral and ventral lines observed in *A*. *oxyrinchus* is lesser than observed in *A*. *sturio* [30]. Dorsal scutes are more numerous in *A*. *sturio* (usually between 13 and 14) than in *A*. *oxyrinchus* (on average 10) [14]. The sturgeon caught off Gijón had 10 free dorsal scutes (plus an anterior one fused with the dermocranium), the average value for *A*. *oxyrinchus*.

The female sturgeon caught at Gijón is an adult specimen (females mature when they are 27–28 years old, with a total length of 1900 mm) [2]. The age of the fish was not determined, but a total length of 2500 mm may correspond of an age of at least 30 years [46]. The largest reported *A*. *oxyrinchus* was a 4600 mm female weighing 365 kg, which was caught in the St. John River estuary, New Brunswick, in 1924 [23]. The species is long lived, reaching ages in excess of 60 years for females and approximately 30 years for males [2].

The gonadosomatic index of the sturgeon caught at Gijón was very low (1.565), as this index in a reproductive female can reach approximately 25 [2]. Based on this calculation, we cannot consider this female sturgeon to have been in a pre-reproductive season.

### Molecular analyses

The Cyt b sequence of the Spanish specimen is identical to the *A*. *o*. *oxyrinchus* haplotype described by [35] and found in both sides of the Atlantic Ocean [18]. This haplotype is currently dominant in the Canadian and Mid-Atlantic clusters [12]. The Cyt b sequence of our specimen also matches the partial sequence (424 bp) of haplotypes H2 to H5 of *A*. *o*. *oxyrinchus* found in the St. Lawrence, St. John and Hudson rivers, and Baltic medieval populations [18], as well as in haplotypes H7 and H8 of *A*. *o*. *desotoi* from the Gulf of Mexico. The sequence divergence with other *A*. *o*. *oxyrinchus* and *A*. *o*. *desotoi* haplotypes varies between 0.1 and 0.2%. Likewise, the sequence divergence with *A*. *o*. *oxyrinchus* stocked in Polish waters [44] is 0.2%. Finally, the sequence divergence with *A*. *sturio* from the Guadalquivir and Gironde rivers ranged from 3.3 to 4.3% (S2 Table).

The entire D-loop sequence of the specimen matches the short fragment described as the haplotype A of *A*. *o*. *oxyrinchus*, found in both sides of the Atlantic Ocean. This haplotype is common in the western Atlantic (Canadian, Mid-Atlantic and Southeastern clusters [12]), United Kingdom, North Sea, and modern and archaeological Baltic Sea records. Furthermore, the haplotype has from one to six bp changes with different haplotypes described throughout the range of *A*. *o*. *oxyrinchus*. These changes correspond with a divergence between 0.6 and 3.5%. The differences with *A*. *o*. *desotoi* haplotypes ranged from five to eight changes (divergence between 2.9 and 4.6%), and 43 changes with *A*. *sturio* (divergence of 28.4%) (S3 Table).

### Taxonomy

*Acipenser sturio* was considered for long time to be the only sturgeon species native to southwestern Europe [47–50]. The eventual occurrence of *A*. *naccarii* in Spain was then suggested [51], but that conclusion was refuted by means of morphology [8, 28, 52–54] and molecular analyses [55–57]. Additional molecular data supporting the possible occurrence of *A*. *naccarii* in Spain were presented [58–59], but the distribution range of the species outside the Adriatic Sea was not further confirmed [4, 14–15, 26, 60–62].

Morphological and molecular characters of archaeological samples of Iberian sturgeons, covering a time frame from ~11,000 years ago up to the 15th century, were analyzed, and all specimens were determined to be *A*. *sturio*, whereas no mitochondrial haplotypes of *A*. *naccarii* or *A*. *oxyrinchus* were found [63].

The former last known record of sturgeon in the Spanish waters was a female (Tl = 2100 mm) fished near the Guadalquivir River mouth on 14 September 1992 [49]. In northern Spain, rivers flowing to the Bay of Biscay are small and offer no spawning areas for sturgeons. However, the presence of *A*. *sturio* in the 20th century near to the coast has been proven by captures from the Bay of Biscay, Cantabria, 1914 (Tl = 430 mm), the Bay of Biscay, San Sebastián, Guipúzcoa, 1975 (Tl = 945 mm) and San Vicente de la Barquera, Cantabria, 1988 (Tl = 1205 mm) [52].

The first alleged historic reference of *A*. *oxyrinchus* in Spain comes from one specimen “caught during the eighteenth century in Catalonian rivers, possibly in the Ebro” [59]. This identification was based on molecular analyses, and morphological data of the specimen were not described. This stuffed specimen is kept in the Cabinet Salvador collection of the Botanical Garden of Barcelona and is cataloged with the code number SALV-7509, but according to our inquiries, no original label associated with the specimen exists to confirm its origin in the Ebro River (pers. com. of Neus Ibáñez, curator of the Cabinet Salvador collection). Consequently, the supposed presence of *A*. *oxyrinchus* in the Ebro River in the 18th century [59] is unconfirmed. In fact, *A*. *oxyrinchus* seems to be absent from the French Mediterranean Sea and rivers, where the only proven sturgeon species is *A*. *sturio* [15, 61].

### Origin

A recovery program for *A*. *oxyrinchus* in northern Europe is already in progress. Since 2004, various life cycle stages (fertilized eggs, hatchlings, and fingerlings) obtained through the controlled reproduction of wild spawning *A*. *oxyrinchus* caught in the St. John River in Canada have been imported to three Poland fish culture facilities [64]. More than 70,000 individuals of *A*. *oxyrinchus* (age 0+ to 1+) have been released into the Oder River basin since 2006, and more than 51,000 fish (0+ to 2+) have been released into the Vistula River basin since 2007 [64]. The sturgeon caught near Gijón in 2010 cannot belong to these stocked fishes because its size suggest it is an adult fish at least 30 years old, and consequently, it has a natural origin. Furthermore, the Cyt b gene of the Spanish specimen has a sequence divergence of 0.2% with that of *A*. *oxyrinchus* of Canadian origin stocked in Poland [44].This also supports the natural origin of the sturgeon caught in Spain.

*Acipenser oxyrinchus* exhibits a strong homing behavior, but strays can colonize other river systems. Tagged *A*. *oxyrinchus* move widely through the near Atlantic shore, along the eastern coast of North America. Pop-up satellite transmitters showed that they use waters within approximately 100 km of the coastline during their migration [65–66]. Otherwise, the species is able to undertake long oceanic migrations, up to thousands of kilometers; based on tagging studies, movements of up to 1450 km have been recorded [2]. Thus, vagrant specimens have been collected off Labrador, Bermuda, and Venezuela, in addition to those founded the population from the Baltic Sea [41].

The specimen of *A*. *oxyrinchus* cited in this paper, near Gijón in 2010, was found approximately 900 km from the last known specimen of the same species caught in 2004 off the Welsh coast [20], and approximately 2500 km from one captured near the Estonian coast in 1996 [67]. In addition, the report of *A*. *oxyrinchus* in northern Spain is more than 4000 km from the Gulf of St. Lawrence in North America.

Based on this background, the following question arises: Should this fish be considered a stray, migrant or vagrant? Because the migratory movements of this species in the open ocean are not well known yet, it would be preferable to consider the sturgeon of Gijón as a stray or vagrant specimen. Given the high molecular similarity between the Baltic Sea stocks and northernmost populations of North America, its eventual birthplace could not be determined.

### Conservation

After the new record of *A*. *oxyrinchus* reported herein, three sturgeon species can be found in southwestern Europe: *A*. *sturio* in the Garonne River basin in France and adjacent seas, *A*. *naccarii* in the Po River basin in Italy, and *A*. *oxyrinchus* with the two most recent reports, one from the coast of Wales in 2004 and one from the coast of northern Spain in 2010. No available river is known as a possible breeding of the last species in the area.

*Acipenser sturio* is categorized as Critically Endangered, criteria A2cde;B2ab(ii,iii,v) [25]. The current size of the Garonne population is approximately 20–750 native wild adult fish [25]. Recovery efforts for this species have been implemented in France and Germany since the early 1990s and in the Netherlands since the 2000s [68–70]. Consequently, reintroduction programs are currently in progress in the Garonne-Dordogne, Rhine and Elbe rivers.

*Acipenser naccarii* is categorized as Critically Endangered, criteria A2bcde;B2ab(i,ii,iii,iv,v) [26]. A recovery plan for this species has been implemented by several public administrations in Italy since the early 1990s, with scientific research and restocking actions that involved the reintroduction of nearly a half million specimens of different sizes to the Po River. However, there is no evidence to confirm continuing reproduction in the wild [26].

Recent evidence from archaeological remains show that it is unlikely that any functional population of *A*. *naccarii* existed apart from the Adriatic Sea and particularly in the western Mediterranean [4, 14–16, 61–63]. In fact, records of *A*. *naccarii* from the Tyrrhenian slope of Italy, Spain and France are considered erroneous [22].

*Acipenser oxyrinchus* is considered Near Threatened globally [71]; but extirpated in Europe due to massive overfishing, damming, river regulation and pollution, where the decline began in 19th century, perhaps even earlier [22]. The number of mature individuals in the North American population is likely considerably more than 10,000 [71].

The Baltic Marine Environment Protection Commission (HELCOM), an intergovernmental organization governing the Convention on the Protection of the Marine Environment of the Baltic Sea Area (Helsinki Convention), considered *A*. *oxyrinchus* as Regionally Extinct (RE) in the area [72]. In Europe, at a national level, only the contracting parties of HELCOM consider the *A*. *oxyrinchus* species as such for conservation purposes. However, other European countries with historic or recent occurrence of *A*. *oxyrinchus* (France, the Netherlands, UK and Spain) need to catalog the species in their respective red lists. As a whole, the residual European stocks of *A*. *oxyrinchus* ought to be listed as Critically Endangered (CR) according to the IUCN categories, as is currently done with *A*. *sturio*.

*Acipenser sturio* is protected under a number of international and European legislations, as well as under national legislation in most countries of its historic range. Meanwhile, European surviving specimens or populations of *A*. *oxyrinchus* may actually be unprotected by European and national regulations because the species as such is not cataloged in many countries.

In listing the single scientific name *A*. *sturio* for the sturgeon, the EC Habitats Directive enshrined into law a nomenclature based on taxonomic opinion as it stood in 1992. Today, two species must be treated under the one heading: *A*. *sturio* and *A*. *oxyrinchus*. Direct commercial fishing of sturgeons is prohibited by the EU fisheries regulations, but all the by-caught specimens should be immediately returned to the sea. Every year, several Atlantic sturgeon specimens are lost, as fishermen are not aware of the species' legal status and of the instructions to be followed if caught. Unfortunately, the catch and landing of the sturgeon in Gijón in 2010 is the last in a series of recent by-catch incidents of large Atlantic sturgeons (*A*. *sturio* or *A*. *oxyrinchus*) in several European countries, such as occurred in Leiden, the Netherlands, in 2003; Swansea, UK, in 2004; Les Sables d'Olonne, France, in 2004, and Ijmuiden, the Netherlands, in 2007. Information campaigns with the fishermen, have already been implemented in France for *A*. *sturio*, but are needed for both species in the concerned countries, which have their fishing fleets working in southwestern Europe (Belgium, Denmark, France, Germany, Ireland, the Netherlands, Portugal, Spain and UK), to effectively protect the remaining Atlantic sturgeon.

A recovery action for the Baltic *A*. *oxyrinchus* population has been recently developed by cooperation between Germany and Poland. Brood stocks from the St. John River, Canada, were transferred to Europe in 2006 and 2007, and experimental releases of reared juvenile *A*. *oxyrinchus* have been carried out annually since 2006 in the Oder and Vistula rivers [64, 73]. These ongoing re-introduction programs have not yet resulted in any known successful reproduction in the wild.

Furthermore, the possible co-occurrence of *A*. *sturio* and *A*. *oxyrinchus* raises new challenges for sturgeon conservation in Europe. For instance, new key questions concern the accurate selection of species selection for restoration program in southwestern European countries. In fact, the reintroduction of *A*. *oxyrinchus* in French waters was suggested [74]. However, a proposal has also been made to protect the extant fragile Gironde population of *A*. *sturio* from contacts with *A*. *oxyrinchus* and to restrict *A*. *oxyrinchus* reintroduction to the Baltic region, despite its historical presence on the French coast [75].

The viability of the scarce surviving populations of European Atlantic sturgeons is subject to climate change and current global warming, especially along the southern limit of their range [76], which may have particular impact given the different thermal preferences of the two species [40].

Finally, it is recommended that more inclusive and, therefore, accurate monitoring take place in southwestern Europe to obtain appropriate information for the assessment of the occurrence of both species of Atlantic sturgeon. Knowledge about movements and the spatial distribution of sturgeon specimens can help in the improvement of effective recovery plans [77].

## Supporting Information

S1 Fig Allele size values of the microsatellite loci. Allele sizes (numbers of bp with arrows) found in the sturgeon specimen caught off the coast of Gijón in 2010, compared with the known size ranges of five microsatellite loci (*LS19*, *LS54*, *LS68*, *Aox23* and *AoxD161*) used to distinguish *A*. *oxyrinchus*, *A*. *sturio* and *A*. *naccarii*.
(DOCX)

S1 Table Morphometric and meristic characters of the sturgeon specimen caught off the coast of Gijón in 2010. (DOCX)

S2 Table Comparative analysis of the complete Cyt b sequence (1139 bp) of the sturgeon specimen caught off the coast of Gijón in 2010 with available sequences of *A*. *oxyrinchus* and *A*. *sturio* retrieved from GenBank. GenBank: accession number in GenBank; Region: Regional clusters in the western Atlantic [1]: Gulf (*A*. *o*. *desotoi* in the tributaries of the Gulf of Mexico), Southeastern (rivers in Georgia and South Carolina), Mid-Atlantic (Hudson and Delaware rivers), Canadian (Kennebec, St. Lawrence and St. John); bp: base pair; genetic distance: pairwise distances following Kimura’s two-parameter model.
(DOCX)

S3 Table Comparative analysis of the complete D-loop sequence (880 bp) of the sturgeon specimen caught off the coast of Gijón in 2010 with available sequences of *A*. *oxyrinchus* and *A*. *sturio* retrieved from GenBank. GenBank: accession number in GenBank; Region: Regional clusters in the western Atlantic [1]: Gulf (*A*. *o*. *desotoi* in the tributaries of the Gulf of Mexico), Southeastern (rivers in Georgia and South Carolina), Mid-Atlantic (Hudson and Delaware rivers), Canadian (Kennebec, St. Lawrence and St. John); bp: base pair; genetic distance: pairwise distances following Kimura’s two-parameter model.
(DOCX)
