# Supplementary material for: Current Occurrence of the Atlantic Sturgeon Acipenser oxyrinchus in Northern Spain: A New Prospect for Sturgeon Conservation in Western Europe
Source: PLoS One. 2015 Dec 30;10(12):e0145728. doi: 10.1371/journal.pone.0145728 (PMC4696671; doi:10.1371/journal.pone.0145728)

**S1 Fig. Allele size values of the microsatellite loci.** Allele sizes (numbers of bp with arrows) found in the sturgeon specimen caught off the coast of Gijón in 2010, compared with the known size ranges of five microsatellite loci (*LS19*, *LS54*, *LS68*, *Aox23* and *AoxD161*) used to distinguish *A. oxyrinchus*, *A. sturio* and *A. naccarii*.


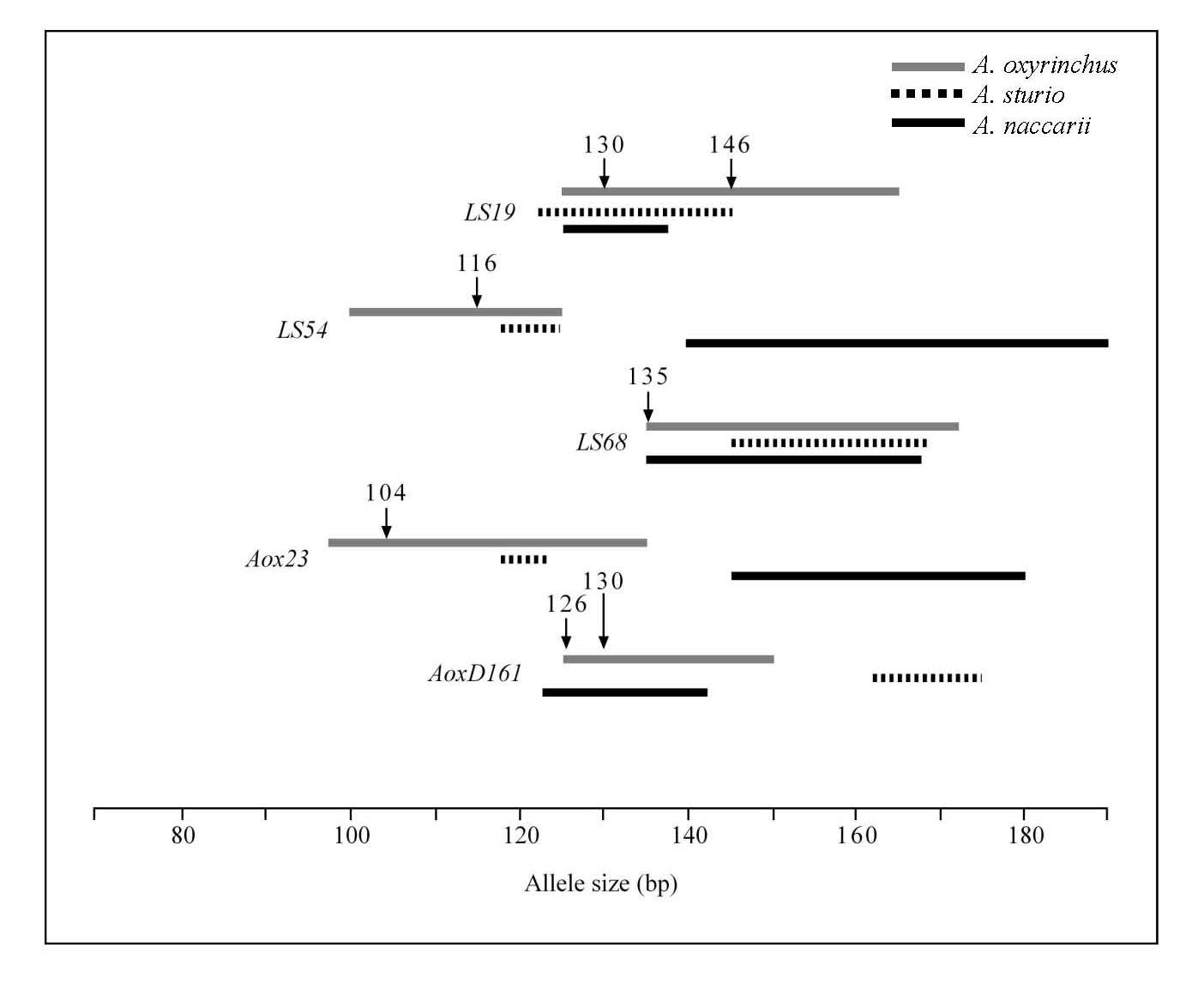

Supplement: S1 Fig — Allele sizes (numbers of bp with arrows) found in the sturgeon specimen caught off the coast of Gijón in 2010, compared with the known size ranges of five microsatellite loci (LS19, LS54, LS68, Aox23 and AoxD161) used to distinguish A. oxyrinchus, A. sturio and A. naccarii. (DOCX) [file pone.0145728.s001.docx]
