# Supplementary material for: Current Occurrence of the Atlantic Sturgeon Acipenser oxyrinchus in Northern Spain: A New Prospect for Sturgeon Conservation in Western Europe
Source: PLoS One. 2015 Dec 30;10(12):e0145728. doi: 10.1371/journal.pone.0145728 (PMC4696671; doi:10.1371/journal.pone.0145728)
Supplement: S1 Table — (DOCX) [file pone.0145728.s002.docx]

**S1 Table. Morphometric and meristic characters of the sturgeon specimen caught off the coast of Gijón in 2010.**

| Morphometric measurement | Abbreviation | (mm) |  | Meristic count | Abbreviation |  |
| --- | --- | --- | --- | --- | --- | --- |
| Total length | Tl | 2500 |  | Gill rakers (left) | Sp.br.-left | 18 |
| Fork length | Fl | 2285 |  | Gill rakers (right) | Sp.br.-right | 18 |
| Standard length | Sl | 2205 |  | Dorsal scutes | SD | (1)10 |
| Length of head | lc | 480 |  | Lateral scutes (left) | SL-left | 27 |
| Preorbital distance | prO | 170 |  | Lateral scutes (right) | SL-right | 27 |
| Horizontal diameter of eye | Oh | 26 |  | Ventral scutes (left) | SV-left | 9 |
| Postorbital distance | poO | 285 |  | Ventral scutes (right) | SV-right | 9 |
| Predorsal distance | pD | 1755 |  | Post-dorsal fin plates |  | 2+2 |
| Prepectoral distance | pP | 500 |  | Post-anal plates |  | 2+1 |
| Preventral distance | pV | 1510 |  | Post-anal fin plates |  | 1+2+1 |
| Preanal distance | pA | 1810 |  |  |  |  |
| Length of caudal peduncle from dorsal fin | lpcd | 130 |  |  |  |  |
| Length of caudal peduncle from anal fin | lpc | 120 |  |  |  |  |
| Distance between pectoral and ventral fins | P-V | 900 |  |  |  |  |
| Distance between pectoral and anal fins | P-A | 1210 |  |  |  |  |
| Distance between ventral and anal fins | V-A | 205 |  |  |  |  |
| Length of dorsal fin | lD | 200 |  |  |  |  |
| Depth of dorsal fin | hD | 230 |  |  |  |  |
| Length of pectoral fin base | lPbs | 110 |  |  |  |  |
| Length of pectoral fin | lP | 345 |  |  |  |  |
| Length of ventral fin base | lVbs | 105 |  |  |  |  |
| Length of ventral fin | lV | 195 |  |  |  |  |
| Length of anal fin base | lA | 145 |  |  |  |  |
| Depth of anal fin | hA | 255 |  |  |  |  |
| Maximum body depth | H | 350 |  |  |  |  |
| Minimum body depth | h | 85 |  |  |  |  |
| Head depth at center of eye | hco | 155 |  |  |  |  |
| Head depth at nape | hc | 280 |  |  |  |  |
| Head with | lac | 260 |  |  |  |  |
| Interorbital distance | io | 190 |  |  |  |  |
