# Supplementary material for: Current Occurrence of the Atlantic Sturgeon Acipenser oxyrinchus in Northern Spain: A New Prospect for Sturgeon Conservation in Western Europe
Source: PLoS One. 2015 Dec 30;10(12):e0145728. doi: 10.1371/journal.pone.0145728 (PMC4696671; doi:10.1371/journal.pone.0145728)
Supplement: S2 Table — GenBank: accession number in GenBank; Region: Regional clusters in the western Atlantic [1]: Gulf (A. o. desotoi in the tributaries of the Gulf of Mexico), Southeastern (rivers in Georgia and South Carolina), Mid-Atlantic (Hudson and Delaware rivers), Canadian (Kennebec, St. Lawrence and St. John); bp: base pair; genetic distance: pairwise distances following Kimura’s two-parameter model. (DOCX) [file pone.0145728.s003.docx]

**S2 Table. Comparative analysis of the complete Cyt b sequence (1139 bp) of the sturgeon specimen caught off the coast of Gijón in 2010 with available sequences of *A. oxyrinchus* and *A. sturio* retrieved from GenBank.** GenBank: accession number in GenBank; Region: Regional clusters in the western Atlantic [1]: Gulf (*A. o. desotoi* in the tributaries of the Gulf of Mexico), Southeastern (rivers in Georgia and South Carolina), Mid-Atlantic (Hudson and Delaware rivers), Canadian (Kennebec, St. Lawrence and St. John); bp: base pair; genetic distance: pairwise distances following Kimura’s two-parameter model.

| **Species** | **GenBank** | **Reference** | **Haplotype** | **Region** | **bp number** | **bp changes** | **genetic distance** |
| --- | --- | --- | --- | --- | --- | --- | --- |
| *A. oxyrinchus oxyrinchus* | AJ245838 | [2] | - | Canadian, Mid-Atlantic | 1170 | 0 | 0.000 |
| *A. oxyrinchus oxyrinchus* | JX669882 | [3] | Aoo_AOH_004 | - | 806 | 0 | 0.000 |
| *A. oxyrinchus oxyrinchus* | - | [3] | H2 | Baltic, Canadian, Mid-Atlantic | 424 | 0 | 0.000 |
| *A. oxyrinchus oxyrinchus* | - | [3] | H3 | Canadian, Mid-Atlantic | 424 | 0 | 0.000 |
| *A. oxyrinchus oxyrinchus* | - | [3] | H4 | Mid-Atlantic | 424 | 0 | 0.000 |
| *A. oxyrinchus oxyrinchus* | - | [3] | H5 | Mid-Atlantic | 424 | 0 | 0.000 |
| *A. oxyrinchus desotoi* | - | [3] | H7 | Gulf | 424 | 0 | 0.000 |
| *A. oxyrinchus desotoi* | - | [3] | H8 | Gulf | 424 | 0 | 0.000 |
| *A. oxyrinchus desotoi* | JX669880 | [3] | Gulf_001 | Gulf | 806 | 1 | 0.001 |
| *A. oxyrinchus oxyrinchus* | - | [3] | H1 | Baltic | 424 | 1 | 0.002 |
| *A. oxyrinchus desotoi* | - | [3] | H6 | Gulf | 424 | 1 | 0.002 |
| *A. oxyrinchus oxyrinchus* | KC987018 | [4] | - | Canadian, stocked in Poland | 344 | 1 | 0.002 |
| *A. oxyrinchus desotoi* | JX669881 | [3] | Gulf_018 | Gulf | 806 | 2 | 0.002 |
| *A. sturio* | AF217209 | [5] | - | Spain | 155 | 13 | 0.033 |
| *A. sturio* | - | [3] | H9 | France | 424 | 15 | 0.037 |
| *A. sturio* | AJ245839 | [2] | - | France | 1170 | 47 | 0.043 |
