# Supplementary material for: Current Occurrence of the Atlantic Sturgeon Acipenser oxyrinchus in Northern Spain: A New Prospect for Sturgeon Conservation in Western Europe
Source: PLoS One. 2015 Dec 30;10(12):e0145728. doi: 10.1371/journal.pone.0145728 (PMC4696671; doi:10.1371/journal.pone.0145728)
Supplement: S3 Table — GenBank: accession number in GenBank; Region: Regional clusters in the western Atlantic [1]: Gulf (A. o. desotoi in the tributaries of the Gulf of Mexico), Southeastern (rivers in Georgia and South Carolina), Mid-Atlantic (Hudson and Delaware rivers), Canadian (Kennebec, St. Lawrence and St. John); bp: base pair; genetic distance: pairwise distances following Kimura’s two-parameter model. (DOCX) [file pone.0145728.s004.docx]

**S3 Table. Comparative analysis of the complete D-loop sequence (880 bp) of the sturgeon specimen caught off the coast of Gijón in 2010 with available sequences of *A. oxyrinchus* and *A. sturio* retrieved from GenBank.** GenBank: accession number in GenBank; Region: Regional clusters in the western Atlantic [1]: Gulf (*A. o. desotoi* in the tributaries of the Gulf of Mexico), Southeastern (rivers in Georgia and South Carolina), Mid-Atlantic (Hudson and Delaware rivers), Canadian (Kennebec, St. Lawrence and St. John); bp: base pair; genetic distance: pairwise distances following Kimura’s two-parameter model.

| **Species** | **GenBank** | **Reference** | **Haplotype** | **Region** | **bp number** | **bp changes** | **genetic distance** |
| --- | --- | --- | --- | --- | --- | --- | --- |
| *A. oxyrinchus oxyrinchus* | AF162716 | [6], [7], [8], [9] | A | Canadian, Mid-Atlantic, Southeastern | 203 | 0 | 0.000 |
| *A. oxyrinchus oxyrinchus* | AJ249670 | [1], [2], [10], [11] | A | Canadian, Mid-Atlantic, Southeastern, Baltic (archaeological, modern ), UK | 245 | 0 | 0.000 |
| *A. oxyrinchus oxyrinchus* | H1 | [3] | H1 | Baltic (pre-Roman, medieval, late medieval, modern) | 208 | 0 | 0.000 |
| *A. oxyrinchus oxyrinchus* | H2 | [3] | H2 | Baltic (medieval) | 208 | 0 | 0.000 |
| *A. oxyrinchus oxyrinchus* | AF162717 | [6], [7], [8], [9] | A1 | Canadian | 203 | 1 | 0.006 |
| *A. oxyrinchus oxyrinchus* | AF162718 | [6], [7], [8] | A2 | Mid-Atlantic, Southeastern | 203 | 1 | 0.006 |
| *A. oxyrinchus oxyrinchus* | AF162719 | [6], [7], [8], [9] | A3 | Southeastern | 203 | 1 | 0.006 |
| *A. oxyrinchus oxyrinchus* | AF162720 | [6], [7], [8] | A4 | Southeastern | 203 | 1 | 0.006 |
| *A. oxyrinchus oxyrinchus* | AF162721 | [6], [7], [8], [9] | B | Mid-Atlantic | 203 | 1 | 0.006 |
| *A. oxyrinchus oxyrinchus* | AF162729 | [6], [7], [8], [9] | E | Southeastern | 203 | 1 | 0.006 |
| *A. oxyrinchus oxyrinchus* | AF162732 | [6], [7], [8], [9] | N | Mid-Atlantic, Southeastern | 203 | 1 | 0.006 |
| *A. oxyrinchus oxyrinchus* | EU420008 | [11] | Aoo600 | UK | 203 | 1 | 0.006 |
| *A. oxyrinchus oxyrinchus* | EU684143 | [1] | BS1 | Baltic (medieval) | 203 | 1 | 0.006 |
| *A. oxyrinchus oxyrinchus* | EU726274 | [8], [9] | A5 | Mid-Atlantic | 203 | 1 | 0.006 |
| *A. oxyrinchus oxyrinchus* | H3 | [3] | H3 | Canadian, Mid-Atlantic | 208 | 1 | 0.006 |
| *A. oxyrinchus oxyrinchus* | AF162722 | [6], [7], [8], [9] | C | Mid-Atlantic, Southeastern | 203 | 2 | 0.011 |
| *A. oxyrinchus oxyrinchus* | AF162730 | [6], [7], [8] | E1 | Southeastern | 203 | 2 | 0.011 |
| *A. oxyrinchus oxyrinchus* | AF162731 | [6], [7], [8] | E2 | Southeastern | 203 | 2 | 0.011 |
| *A. oxyrinchus oxyrinchus* | AF162733 | [6], [7], [8], [9] | N1 | Mid-Atlantic, Southeastern | 203 | 2 | 0.011 |
| *A. oxyrinchus oxyrinchus* | AF162734 | [6], [7], [8], [9] | N2 | Southeastern | 203 | 2 | 0.011 |
| *A. oxyrinchus oxyrinchus* | AF162738 | [6], [7], [8] | P2 | Southeastern | 203 | 2 | 0.011 |
| *A. oxyrinchus oxyrinchus* | AF162749 | [6], [7], [8] | E3 | Southeastern | 203 | 2 | 0.011 |
| *A. oxyrinchus oxyrinchus* | AF162752 | [6], [7], [8] | T | Southeastern | 203 | 2 | 0.011 |
| *A. oxyrinchus oxyrinchus* | AF162753 | [6], [7], [8], [9] | B1 | Mid-Atlantic | 203 | 2 | 0.011 |
| *A. oxyrinchus oxyrinchus* | EU684144 | [1] | BS2 | Baltic (medieval) | 203 | 2 | 0.011 |
| *A. oxyrinchus oxyrinchus* | EU726275 | [9] | B2 | Mid-Atlantic | 557 | 2 | 0.011 |
| *A. oxyrinchus oxyrinchus* | AF162723 | [6], [7], [8], [9] | C1 | Mid-Atlantic, Southeastern | 203 | 3 | 0.017 |
| *A. oxyrinchus oxyrinchus* | AF162724 | [6], [7], [8], [9] | C2 | Southeastern | 203 | 3 | 0.017 |
| *A. oxyrinchus oxyrinchus* | AF162735 | [6], [7], [8], [9] | O | Southeastern | 203 | 3 | 0.017 |
| *A. oxyrinchus oxyrinchus* | AF162737 | [6], [7], [8], [9] | P1 | Southeastern | 203 | 3 | 0.017 |
| *A. oxyrinchus oxyrinchus* | AF162739 | [6], [7], [8] | P3 | Southeastern | 203 | 3 | 0.017 |
| *A. oxyrinchus oxyrinchus* | AF162740 | [6], [7], [8] | P4 | Southeastern | 203 | 3 | 0.017 |
| *A. oxyrinchus oxyrinchus* | AF162745 | [6], [7], [8], [9] | P9 | Southeastern | 203 | 3 | 0.017 |
| *A. oxyrinchus oxyrinchus* | AF162746 | [6], [7], [8], [9] | P10 | Southeastern | 203 | 3 | 0.017 |
| *A. oxyrinchus oxyrinchus* | AF162750 | [6], [7], [8] | S | Southeastern | 203 | 3 | 0.017 |
| *A. oxyrinchus oxyrinchus* | AF162754 | [6], [7], [8] | U | Southeastern | 203 | 3 | 0.017 |
| *A. oxyrinchus oxyrinchus* | AF162726 | [6], [7], [8], [9] | D | Mid-Atlantic, Southeastern | 203 | 4 | 0.023 |
| *A. oxyrinchus oxyrinchus* | AF162736 | [6], [7], [8], [9] | P | Mid-Atlantic, Southeastern | 203 | 4 | 0.023 |
| *A. oxyrinchus oxyrinchus* | AF162741 | [6], [7], [8], [9] | P5 | Southeastern | 203 | 4 | 0.023 |
| *A. oxyrinchus oxyrinchus* | AF162748 | [6], [7], [8], [9] | R | Mid-Atlantic | 203 | 4 | 0.023 |
| *A. oxyrinchus oxyrinchus* | AF162751 | [6], [7], [8], [9] | S1 | Mid-Atlantic, Southeastern | 203 | 4 | 0.023 |
| *A. oxyrinchus oxyrinchus* | EU726276 | [8], [9] | C4 | Mid-Atlantic | 557 | 4 | 0.023 |
| *A. oxyrinchus oxyrinchus* | EU726278 | [9] | C6 | Southeastern | 557 | 4 | 0.023 |
| *A. oxyrinchus oxyrinchus* | EU726279 | [9] | P12 | Southeastern | 557 | 4 | 0.023 |
| *A. oxyrinchus oxyrinchus* | H4 | [3] | H4 | Mid-Atlantic | 208 | 4 | 0.023 |
| *A. oxyrinchus oxyrinchus* | AF162725 | [6], [7], [9] | C3 | Southeastern | 203 | 5 | 0.029 |
| *A. oxyrinchus oxyrinchus* | AF162727 | [6], [7], [8] | D1 | Southeastern | 203 | 5 | 0.029 |
| *A. oxyrinchus oxyrinchus* | AF162728 | [6], [7], [8], [9] | D2 | Mid-Atlantic, Southeastern | 203 | 5 | 0.029 |
| *A. oxyrinchus oxyrinchus* | AF162742 | [6], [7], [8] | P6 | Southeastern | 203 | 5 | 0.029 |
| *A. oxyrinchus oxyrinchus* | AF162744 | [6], [7], [8], [9] | P8 | Mid-Atlantic, Southeastern | 203 | 5 | 0.029 |
| *A. oxyrinchus oxyrinchus* | AF162747 | [6], [7], [8] | Q | Southeastern | 203 | 5 | 0.029 |
| *A. oxyrinchus oxyrinchus* | EU726277 | [8], [9] | C5 | Mid-Atlantic | 557 | 5 | 0.029 |
| *A. oxyrinchus oxyrinchus* | H5 | [3] | H5 | Mid-Atlantic | 208 | 5 | 0.029 |
| *A. oxyrinchus desotoi* | H6 | [3] | H6 | Gulf | 208 | 5 | 0.029 |
| *A. oxyrinchus oxyrinchus* | AF162743 | [6], [7], [8], [9] | P7 | Southeastern | 203 | 6 | 0.035 |
| *A. oxyrinchus desotoi* | H7 | [3] | H7 | Gulf | 208 | 7 | 0.040 |
| *A. oxyrinchus desotoi* | H8 | [3] | H8 | Gulf | 208 | 8 | 0.046 |
| *A. sturio* | H9 | [3] | H9 | France | 208 | 43 | 0.284 |

**Supporting information references:**

1. Ludwig A, Arndt U, Lippold S, Benecke N, Debus L, King TL, et al. Tracing the first steps of American sturgeon pioneers in Europe. BMC Evol Biol. 2008;8:221. doi: 10.1186/1471-2148-8-221
2. Ludwig A, May MB, Debus L, Jenneckens I. Heteroplasmy in the mtDNA control region of sturgeon (*Acipenser*, *Huso* and *Scaphirhynchus*). Genetics. 2000;156:1933–47.
3. Popovic D, Panagiotopoulou H, Baca M, Stefaniak K, Mackiewicz P, Makowiecki D, et al. The history of sturgeon in the Baltic Sea. J Biogeogr. 2014;41:1590–602. doi: 10.1111/jbi.12307
4. Fopp-Bayat D, Kucinski M, Liszewski T, Teodorowicz T, Lączynska B, Lebeda I. Genetic protocol of Atlantic sturgeon *Acipenser oxyrinchus* (L.) fry for restocking the Vistula river, Poland. J Surv Fish Sci. 2015;2:1–10. doi: 10.18331/SFS2015.2.1.1
5. Almodóvar A, Machordom A, Suárez J. Preliminary results from characterization of the Iberian Peninsula sturgeon based on analysis of the mtDNA cytochrome *b*. Bol Inst Esp Oceanogr. 2000;16:17–27.
6. Wirgin I, Waldman JR, Rosko J, Gross R, Collins MR, Rogers SG, et al. Genetic structure of Atlantic sturgeon populations based on mitochondrial DNA control region sequences. T Am Fish Soc. 2000;129:476–86.
7. Wirgin I, Waldman JR, Stabile J, King T. Comparison of mitochondrial DNA control region sequence and microsatellite DNA analyses in estimating population structure and gene flow rates in Atlantic sturgeon *Acipenser oxyrinchus*. J Appl Ichthyol. 2002;18:313–9.
8. Wirgin I, Grunwald C, Stabile J, Waldman J. Genetic evidence for relict Atlantic sturgeon stocks along the mid-Atlantic coast of the USA. N Am J Fish Manage. 2007;27:1214–29. doi: 10.1577/M06-269.1
9. Grunwald C, Maceda L, Waldman J, Stabile J, Wirgin I. Conservation of Atlantic sturgeon *Acipenser oxyrinchus oxyrinchus*: delineation of stock structure and distinct population segments. Conserv Genet. 2008;9:1111–24. doi: 10.1007/s10592-007-9420-1
10. Ludwig A, Debus L, Lieckfeldt D, Wirgin I, Benecke N, Jenneckens I, et al. When the American sea sturgeon swam east – a colder Baltic Sea greeted this fish from across the Atlantic Ocean in the Middle Ages. Nature 2002;419:447–8.
11. Ludwig A, Makoviecki D, Benecke N. Further evidence of trans-Atlantic colonization of Western Europe by American Atlantic sturgeons. Archaeofauna. 2009;18:185–92.
